# Burden of eye disease and demand for care in the Bangladesh Rohingya displaced population and host community: A cohort study

**DOI:** 10.1371/journal.pmed.1003096

**Published:** 2020-03-31

**Authors:** Munir Ahmed, Noelle Whitestone, Jennifer L. Patnaik, Mohammad Awlad Hossain, Lutful Husain, Mohammed Alauddin, Mushfiqur Rahaman, David Hunter Cherwek, Nathan Congdon, Danny Haddad

**Affiliations:** 1 Orbis Bangladesh, Dhaka, Bangladesh; 2 Independent consultant, Orbis International, New York, New York, United States of America; 3 Orbis International, New York, New York, United States of America; 4 Department of Ophthalmology, University of Colorado School of Medicine, Aurora, Colorado, United States of America; 5 Cox’s Bazar Baitush Sharaf Hospital, Cox’s Bazar, Bangladesh; Johns Hopkins University Bloomberg School of Public Health, UNITED STATES

## Abstract

**Background:**

There is a growing awareness that addressing chronic as well as acute health conditions may contribute importantly to the well-being of displaced populations, but eye care service has generally not been prioritized in crisis situations. We describe a replicable model of eye care provision as delivered by Orbis International and local partners to the Rohingya and host population in Cox’s Bazar, Bangladesh, and characterize the burden of vision impairment and demand for sight-restoring services in this setting.

**Methods and findings:**

Orbis International and local secondary facility Cox’s Bazar Baitush Sharaf Hospital (CBBSH) provide eye care support to the Rohingya population and the host community of all ages in Cox’s Bazar, Bangladesh, with fixed vision screening locations established in Camps 4 and 11 of the Kutupalong refugee settlement. Structured outreach targets these camps and four surrounding local subdistricts, with referrals made as needed for refraction (glasses measurement) and cataract surgery to CBBSH. Between February 2018 and March 2019, 48,105 displaced Rohingya (70.3%, among whom 71.6% were children and 46.5% women) and 20,357 local residents (29.7%, 88.5% children, 54.4% women) underwent vision screening. Displaced Rohingya sought services from a total of 12 surrounding camps, within which coverage was 17.3%, including 43.3% (27,027/62,424) of children aged 5–11 years and 60.0% (5,315/8,857) of adults ≥ 60 years old. The prevalence of blindness (presenting acuity < 3/60) among Rohingya patients exceeded that among local residents by 3- to 6-fold in each 10-year age group between 18 and 59 years (*P* < 0.001 comparing vision between the two groups in this age range), and the prevalence of cataract requiring surgery was also higher in Rohingya patients (18–29 years: 4.67% versus 1.80%, *P* = 0.0019; 30–39: 7.61% versus 2.39%, *P* < 0.001; and 40–49 years: 7.91% versus 3.77%, *P* = 0.0014). A limitation of the study is lack of data on population prevalence of eye disease.

**Conclusions:**

The burden of untreated eye disease is very high among the Rohingya, particularly those in their peak working years who could contribute most to the resiliency of their community. Demand for eye care service is also great among children and adults in this population with many competing healthcare priorities. Research is needed, building on strong evidence of benefit in settled populations, to explore the specific impact of vision care on the well-being of displaced populations.

## Abstract 2

Danny Hadad et al report the burden of untreated vision impairment among the Rohingya people in Cox's Bazar, Bangladesh.

## Author summary

**Why was this study done?:**

- It is increasingly acknowledged that addressing non-emergent health problems can improve the resilience of displaced communities and that such populations can suffer significant burdens of vision impairment.
- However, few if any studies have systematically assessed the demand for eye care in conflict or crisis situations, and models to deliver vision services in such settings have rarely been documented.

**What did the researchers do and find?:**

- Between February 2018 and March 2019, 48,105 displaced Rohingya and 20,357 local residents underwent vision screening.
- Coverage in the population was 43.3% (27,027/62,424) among children aged 5–11 years and 60.0% (5,315/8,857) among adults ≥ 60 years old.
- Prevalence of blindness (presenting acuity < 3/60) among Rohingya patients exceeded by 3- to 6-fold that among local residents between 18 and 59 years old.

**What do these findings mean?:**

- The burden of untreated eye disease is very high among the Rohingya, particularly those in the peak working years who could contribute most to the resiliency of their community.
- Demand for eye care service is great among children and adults in this population with many competing healthcare priorities.

## Introduction

The United Nations High Commissioner for Refugees (UNHCR) reports that the global number of displaced persons is the highest on record, having reached 70.8 million people as of June 2019 [1]. In view of this, the World Health Organization (WHO) has recently identified delivering health in conflict and crisis as one of 13 “Urgent Health Challenges for the Next Decade” [2].

The needs of displaced populations significantly impact the resources of host communities, placing increased pressure on their services and infrastructure [3]. In response to the growing number of such crises, the global community and responding organizations have understandably focused on the urgent remediation of potentially life-threatening conditions, such as infectious disease outbreaks and malnutrition. There is, however, a growing understanding [4–6] that addressing non-emergent conditions such as mental disorders and various disabilities can improve the resilience of displaced communities and may thus be a crucial part of comprehensive strategies to help them succeed. One such strategy to promote resilience may be treatments to promote eye health, such as provision of cataract surgery, shown to be among the most cost-effective interventions in all of healthcare, ranking, for example, ahead of various malaria treatments and oral rehydration therapy [7].

Visually impaired people bear an increased risk of poverty, yet 80% or more of global visual impairment is treatable or preventable [8], and evidence shows improvement in quality of life and increases in household income following cataract surgery [9]. Progress has been made to reduce unnecessary blindness globally [10] through the efforts of the Vision 2020 global initiative and WHO’s Global Action Plan: 2014–2019 [8]. However, these initiatives have largely focused on the needs of settled populations. A growing body of information exists about the visual health and unique eye care needs of displaced people [11,12], and there is increasing awareness of the unmet need for vision care in these populations.

As a consequence of an outbreak of violence in August 2017, over 742,000 Rohingya, members of a stateless Muslim minority, fled from Myanmar to Bangladesh, making Kutupalong refugee settlement in Cox’s Bazar the world’s largest such facility [3]. Since February 2018, Orbis International and local partners have provided eye care services to the Rohingya in Kutupalong. The current report describes the Orbis model of eye service delivery targeting displaced populations and the host communities supporting them. It also details the burden of need and demand for eye service among the Rohingya and local population as identified in our program between February 2018 and March 2019. It should be noted that these data are clinic-based and are not presented as an unbiased estimate of population disease prevalence in this setting. To the best of our knowledge, this study represents one of the largest published datasets concerning refugee eye health. The model described here is applicable to displaced populations elsewhere and can serve as a template for other organizations doing similar work. We hypothesized that demand for eye care services would be high among the Rohingya and that their burden of correctable vision impairment would be at least as significant as in the host population.

## Methods

### Setting and participants

**Fig 1:**
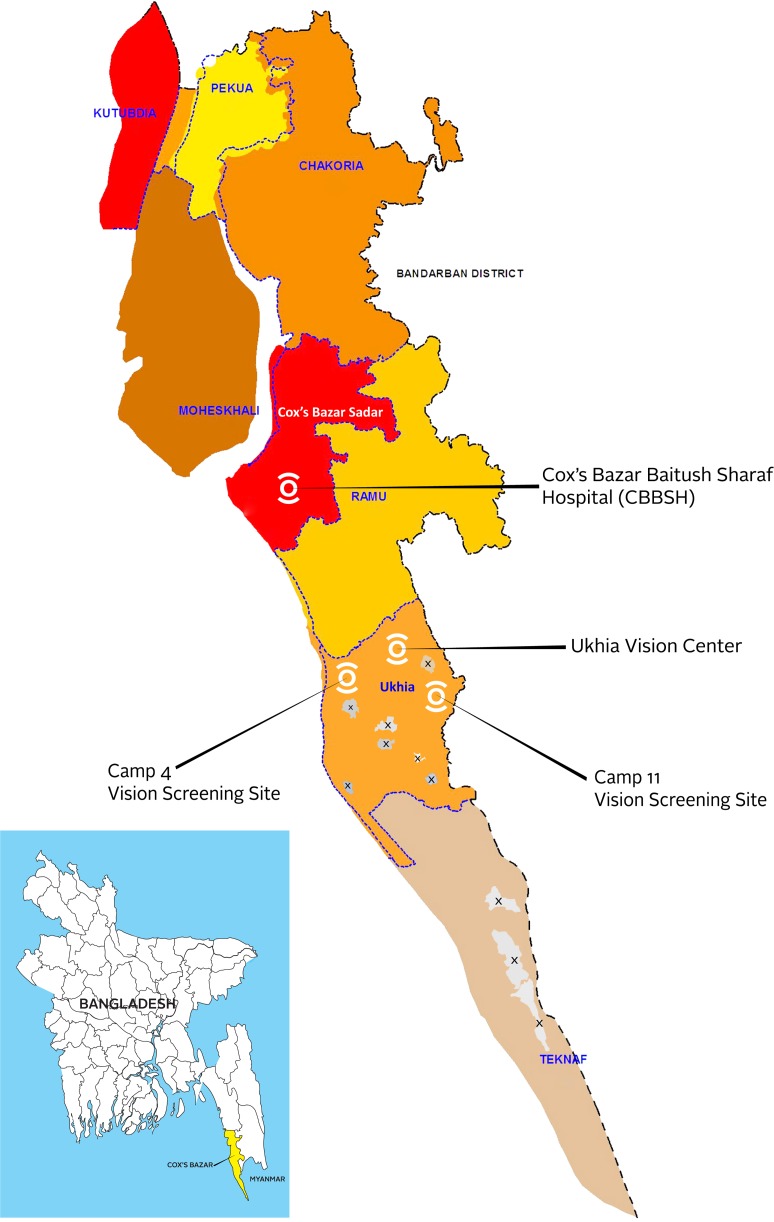
A map of Cox’s Bazaar, showing the study area including vision screening locations. Locations of the Rohingya camps are denoted with shaded Xs. *The base map was obtained from OCHA* *https*:*//www*.*humanitarianresponse*.*info/en/operations/bangladesh/infographic/coxs-bazar-access-road-network-camp-boundary-january-2020*, *CC Attribution 4*.*0*.

Beginning in February 2018 and continuing today, Orbis International has provided eye care support to the Rohingya population and the host community of all ages in Cox’s Bazar, Bangladesh, with vision screening performed in Camps 4 (population 32,389 as of 2019 August) and 11 (population 31,487) [13] of the Kutupalong refugee settlement. Individuals from surrounding camps (population 213,635) [13] are able to participate in the vision screenings. Additionally, there is a structured outreach effort targeting the host-community population of Ukhia (population 207,379 as of 2011 census) and Teknaf (population 264,389), as well as nearby Cox’s Bazar Sadar (population 459,082) and Ramu (population 266,640) subdistricts, all in Cox’s Bazar (Fig 1). Orbis and partner Cox’s Bazar Baitush Sharaf Hospital (CBBSH) implement eye camps, which provide basic screening, together with transportation as needed to CBBSH, a secondary-level facility lying 30 km from the camps, for refraction (measurement for glasses) and provision of spectacles and cataract surgery for children and adults. Program data presented here include Rohingya from a total of 12 camps and the host population of Cox’s Bazar.

The Human Research Ethics Committee at the Asian Institute of Disability and Development (AIDD) deemed the study to be methodologically sound and ethical. AIDD determined that informed consent was not necessary to participate in this research, as the data were collected as part of routine program monitoring and were analyzed in deidentified fashion. AIDD is a disability-focused academic research institute based at the University of South Asia in Dhaka, Bangladesh, and was selected as a reliable and widely respected local center for disability research that could deliver a fair, thorough, and informed ethical review. The study followed the tenets of the Declaration of Helsinki and is reported as per the Strengthening the Reporting of Observational Studies in Epidemiology (STROBE) guideline (S1 STROBE Checklist). The deidentified study dataset is available for download at the Orbis International website (https://www.orbis.org/en/news/2020/rohingya-eye-care-in-numbers?geographicregion=www).

### Vision screening and ocular examination

Vision screenings for the Rohingya population are conducted at the vision center purpose-built for the project in Camp 11, through mobile outreach in Camp 4, and in Child Friendly Spaces in both camps. For the host population, vision screenings are conducted through mobile outreach, at vision centers in subdistrict hospitals, at schools, and at the partner hospital CBBSH. Permission for organizing screening camps is obtained from Government of Bangladesh Camp in Charge (CiC) officials, who disseminate information about screening activities at nongovernmental organization (NGO) coordination meetings. The Orbis outreach team also shares screening dates and locations in advance with Majhi (Rohingya local leaders for a group of 100 families) and Rohingya and host-community religious leaders, along with promotion by loudspeaker. For host-community school screenings, permission is obtained in advance from district/subdistrict education officers and the head teachers of selected schools.

Demographic information is recorded for all presenting adults and children, including whether the individual is a member of the host population or a displaced Rohingya. Ocular examinations are carried out by medical officers or optometrists according to the Standard Cataract Surgery Protocols of National Eye Care Bangladesh [14]. These include vision assessment separately in each eye using a Snellen E chart, examination of the anterior segment by hand light, refraction by a trained refractionist for any eye with presenting vision < 6/12 (performed only during screenings occurring at vision centers or CBBSH), and dispensing of free medications and spectacles as needed. Dilatation of the pupil is performed only on suspected cataract cases (based on hand-light examination) and those requiring a fundus examination (known cases of systemic diabetes, for example) and is done only at CBBSH. Intraocular pressure (IOP) is not routinely measured at the time of the screening eye exam. Children suspected of strabismus are evaluated by an optometrist using the cover-uncover test.

### Management of refractive error

Children and adults with presenting acuity < 6/12 in either eye undergo assessment by a refractionist during screenings occurring at vision centers or at CBBSH, or on referral to these facilities from screenings occurring elsewhere. Spectacles are provided free of charge if needed, with a choice of frame design and colors for children. Cycloplegia is carried out for children < 8 years of age (to relax accommodation and promote accurate results) using cyclopentolate 0.5% one drop every 5 minutes for a total of three drops. Spectacles are also prescribed to patients with visual acuity of 6/9 when symptoms such as headache and asthenopia are present.

### Diagnosis and treatment of cataract

An ophthalmologist at CBBSH performs slit lamp examination of the anterior segment and direct ophthalmoscopy with dilation of the pupil to confirm a suspected diagnosis of cataract on screening. IOP is measured, as are axial length and corneal curvature (to determine the power of the appropriate intraocular lens for cataract surgery) and blood sugar and blood pressure according to the above national Standard Cataract Surgery Protocol [14]. Per protocol, patients with best-corrected visual acuity ≤ 6/36 due to cataract are referred for surgery, though surgery is also available for patients with visual acuity up to 6/18 upon request. Surgery in this program is performed in one eye only in most cases.

### Examination and treatment of pediatric surgical cases

Children failing the screening were further examined at CBBSH. During this project, a child-friendly pediatric outpatient department was constructed at CBBSH for examination of children. All children requiring surgical intervention were referred to the nearby Chittagong Eye Infirmary and Training Complex (CEITC).

### Data collection and management

Identical data collection protocols and software are used for Rohingya and host patients and for adults and children as young as 6 months. Volunteers transcribe patient demographic, diagnostic, and treatment data into the patient information system (PIS) software (custom-built by Orbis Bangladesh, Dhaka, Bangladesh). Data are entered directly into the PIS at the Child Friendly Spaces, vision centers, and the CBBSH partner hospital, within 1 day following any camp or school screening. A password-protected administrative database containing all respondent contact details that is managed by the Orbis Bangladesh office is used for contact purposes and oversight of the data collection process. Paper records of contact sheets, registration documents, and media consent forms are archived under lock and key. All data for research analysis were provided to the statistician (JLP) in a deidentified fashion (with name, contact information, and date of birth removed). The Better Operative Outcome Software Tool (BOOST) application [15] was used to assess visual acuity outcomes on postoperative day 1 after cataract surgery on a random sample of 60 consecutive Rohingya, with vision results benchmarked against a large database of other users according to a previously validated protocol [16].

### Statistical methods

Data were downloaded from PIS into Microsoft Excel 365 (Redmond, WA, United States of America) and imported into SAS version 9.4 (Cary, NC, USA) for statistical analysis. Descriptive analysis included basic frequencies and percentages, with the number of participants indicated who were missing data. Service uptake was calculated as the number of patients included in PIS divided by the total populations of Rohingya and host groups separately. Presenting visual acuity in the better-seeing eye was categorized into four groups: 6/6–6/18, <6/18–6/60, <6/60–3/60, and <3/60. A very small number of patients had two visits, and the better visual acuity was utilized for analyses in the event that recorded visual acuity differed between visits. Prevalence of cataract and refractive error were calculated based on diagnosis present in either or both eyes at one of more examinations. Age stratification was presented using the UNHCR population data’s age groupings for the Rohingya and population census age groupings for the host community. Chi-squared and Fisher exact testing were utilized to determine differences between the Rohingya and host populations for categorical variables, and the Wilcoxon rank sum test was used for continuous variables.

This study did not have a prespecified analytic plan. The only substantive change in our initial statistical approach was the addition of more detailed information on the cohort of postoperative patients undergoing vision assessment using BOOST, including data on preoperative vision. This was done in response to peer reviews, in order to better describe the impact of program-provided surgery on beneficiaries.

## Results

Among 68,462 persons screened by Orbis and local partners between February 2018 and March 2019, 70.3% were displaced Rohingya (*n* = 48,105, 71.6% children, 46.5% women), and 29.7% were local residents (*n* = 20,357, 88.5% children, 54.4% women) from four surrounding upazilas (subdistricts) in Cox’s Bazar. Among this latter group, 9,566 persons (47.0%) reported residing in Ukhia and Teknaf, the two upazilas recognized by the Bangladeshi government as officially comprising the host community, whereas the remainder dwelt in nearby Cox’s Bazar Sadar and Ramu upazilas in Cox’s Bazar. (Both groups are hereafter referred to as “host” communities). Whereas the Orbis screening activities were based in Camps 4 and 11, the Rohingya population presenting for service was drawn additionally from Camps 14, 15, 17, 21, 22, 23, 25, and 27, as well as Kutupalong RC and Nayapara RC camps.

Coverage of vision services among Rohingya dwelling in these 12 camps (48,105/277,511 = 17.3%) was 10 times higher than for the host population (20,357/1,197,490 = 1.70%, *P* < 0.001). (Table 1) Acceptance of vision services among the Rohingya was particularly high among two age groups targeted by the Orbis program as likely to require care: children aged 5–11 years (27,027/62,424 = 43.3%) and adults aged 60 years and above (5,315/8,857 = 60.0%).

**Table 1:** Acceptance of Orbis vision screening among Rohingya living in Camps 4 and 11 and surrounding camps and the host community, stratified by age (note that the age strata differ between Rohingya and host groups in this table, based on the format of available data).

Rohingya Population in Camp 4, Camp 11, and 10 Surrounding Camps				Host Population in 4 Nearby Upazilas (Subdistricts)			
Age (Years)	Screened			Age (Years)	Screened		
	Beneficiaries	Total Population	Coverage (%)		Beneficiaries	Total Population	Coverage (%)
<1	43	8,627	0.50%	0–9	5,078	347,353	1.46%
1–4	1,815	40,623	4.47%	10–19	13,038	288,380	4.52%
5–11	27,027	62,424	43.3%	20–29	509	224,355	0.23%
12–17	5,535	39,479	14.0%	30–39	418	139,236	0.30%
18–59	8,370	117,501	7.12%	40–59	836	141,254	0.59%
60+	5,315	8,857	60.0%	60+	478	56,912	0.84%
Total	48,105	277,511	17.3%	Total	20,357	1,197,490	1.70%

Whereas children aged 5–17 years in the host population had significantly worse-presenting visual acuity than their Rohingya counterparts, the reverse was true for adults in every age category from 18 to 60+ years (*P* < 0.001 for all, Table 2). For example, the prevalence of blindness (<3/60) among the Rohingya exceeded that of the host population by 3- to 6-fold across this entire age range (Table 2). The proportion of women among the blind was significantly higher among the host cohort (57.8%) compared to the Rohingya (44.0%, *P* = 0.030).

**Table 2:** Age-stratified presenting VA in the better-seeing eye among Rohingya and host patients screened in the Orbis outreach program.

Age Group (Years)	Rohingya					Host				
Level of VA logMAR (Snellen)	Total Number	0.0–0.477 (6/6–6/18)	<0.477–1.0 (<6/18–6/60)	<1.0–1.301 (<6/60–3/60)	<1.301 (<3/60)	Total Number	0.0–0.477 (6/6–6/18)	<0.477–1.0 (<6/18–6/60)	<1.0–1.301 (<6/60–3/60)	<1.301 (<3/60)
<1	8	87.5%	0%	0%	12.5%	19	100%	0%	0%	0%
1–4*	1,518	99.5%	0.1%	0%	0.4%	139	99.3%	0.7%	0%	0%
5–11*	26,833	99.8%	0.1%	0%	0.1%	3,675	98.0%	1.7%	0.2%	0.2%
12–17*	5,487	98.9%	0.6%	0.1%	0.4%	9,892	98.0%	1.7%	0.1%	0.2%
18–29**	1,330	89.5%	5.6%	0.8%	4.2%	607	94.4%	3.8%	0.8%	1.0%
30–39**	1,125	85.0%	9.8%	0.9%	4.4%	417	96.6%	2.6%	0.0%	0.7%
40–49**	2,175	74.8%	20.3%	0.9%	4.0%	473	90.7%	8.5%	0.2%	0.6%
50–59**	3,372	61.1%	29.2%	1.6%	8.1%	356	82.3%	14.9%	0.6%	2.2%
60+**	5,047	44.2%	38.2%	3.6%	14.0%	474	65.2%	30.2%	0.6%	4.0%
Total	46,895	89.1%	7.7%	0.6%	2.6%	16,052	96.3%	3.1%	0.2%	0.4%
Missing VA	1,210 (2.52%)					4,305 (21.2%)				

*Presenting logMAR in better-seeing eye is significantly worse for the host population, *P* < 0.001.

**Presenting logMAR in better-seeing eye is significantly worse for the Rohingya population, *P* < 0.001.

Abbreviations: logMAR, log of the minimum angle of resolution; VA, visual acuity (statistical comparisons were made using logMAR values)

The large majority of vision loss in this setting was due to two common conditions, uncorrected refractive error and unoperated cataract, the relative distribution of which differed greatly between the Rohingya and host communities (Table 3). Whereas refractive error (need for glasses) was significantly more common among both host children and adults (except those aged 60+ years), the prevalence of cataract requiring surgery was significantly greater among the Rohingya, with a striking additional burden observed among adults in the peak working years of 18–29 (4.67% versus 1.80%, *P* = 0.0019), 30–39 (7.61% versus 2.39%, *P* < 0.001), and 40–49 (7.91% versus 3.77%, *P* = 0.0014). Cataract was equally common among older patients in both groups, affecting some 14% of those 50–59 years old and nearly 30% aged 60+ years (Table 3).

**Table 3:** Number and diagnosis of patients screened in the Orbis Outreach Program, stratified by age.

Age Group (Years)	Rohingya					Host				
	Patients Screened	Diagnosed With Cataract		Diagnosed With Refractive Error		Patients Screened	Diagnosed With Cataract		Diagnosed With Refractive Error	
	Number	Number	Percent	Number	Percent	Number	Number	Percent	Number	Percent
<1	43	4	9.3%***	0	0%	611	0	0%	0	0%
1–4	1,815	5	0.28%	3	0.17%*	1,772	2	0.11%	10	0.56%
5–11	27,027	32	0.12%*	81	0.30%***	5,117	13	0.25%	354	6.92%
12–17	5,535	19	0.34%***	70	1.26%***	10,513	11	0.10%	1,069	10.2%
18–29	1,391	65	4.67%**	149	10.7%***	612	11	1.80%	118	19.3%
30–39	1,183	90	7.61%***	271	22.9%*	418	10	2.39%	116	27.8%
40–49	2,262	179	7.91%**	1,036	45.8%***	478	18	3.77%	285	59.6%
50–59	3,534	509	14.4%	1,510	42.7%**	358	52	14.5%	182	50.8%
60+	5,315	1,417	26.7%	1,905	35.8%	478	142	29.7%	153	32.0%
Total	48,105	2,320	4.82%***	5,025	10.4%***	20,357	259	1.27%	2,287	11.2%

Please note: No statistical testing for refractive error was performed on the <1-year age group because of row or column sums of zero.

*P ≤ 0.05 comparing Rohingya and host populations.

**P < 0.01 comparing Rohingya and host populations.

***P < 0.001 comparing Rohingya and host populations.

**Fig 2:**
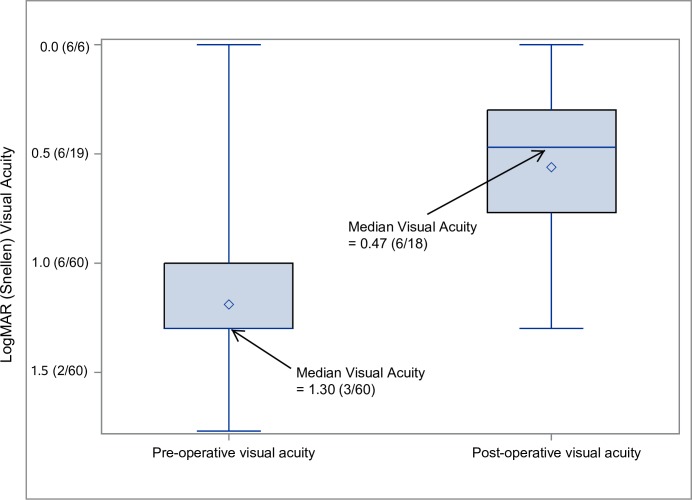
Box plot showing distribution of pre- and postoperative visual acuity for 60 Rohingya patients undergoing cataract surgery in the Orbis program. logMAR, log of the minimum angle of resolution.

The proportion of women among those diagnosed with cataract did not differ between the Rohingya (47.8%) and host groups (52.9%, *P* > 0.05). In the sample of 60 consecutive cataract surgeries performed on Rohingya beneficiaries, 60.0% (*n* = 36) had presenting visual acuity of ≥6/18 on the first postoperative day (WHO definition a good visual result), placing these outcomes in the 74th percentile when benchmarked against a large online database of other users [15,16]. Among this operated sample, 66.7% (*n* = 40) were aged ≥60 years, and 55% (*n* = 33) were women. The median preoperative visual acuity was 1.30 (3/60), and the postoperative value was 0.470 (6/18) (Fig 2).

## Discussion

Our experience in Cox’s Bazar underscores the heavy burden of treatable vision impairment and potential to restore sight with low-cost treatments in this setting. This is not surprising in communities such as the displaced Rohingya, who have been without access to healthcare for a prolonged period [17], and the Cox’s Bazar host population, whose human development index recently ranked last among Bangladesh’s 64 districts [18]. The prevalence of vision impairment and blindness in those presenting to the Orbis clinics was significantly higher among adult Rohingya compared to the host population. The comparatively early age of onset of sight loss in the Rohingya was particularly striking, with much of the additional burden of vision impairment seen during the working years, ages 18 to 59 years.

This phenomenon appears particularly to reflect the high proportion of Rohingya patients presenting with visually significant cataract in their 20s to 40s. Though these figures are clinic- rather than population-based, the presence of some 900 persons under the age of 60 years with visually significant cataract clearly represents a substantial burden to this working-age population. Removal of this burden offers the potential to boost the resilience of their families and communities. Exploring the reasons for the apparent high prevalence of cataract among these young Rohingya patients is beyond the scope of this report, but this may reflect poor access to care and possibly inadequate nutrition, as recent meta-analyses suggest that vitamin supplementation may decrease the risk of lens opacity [19].

Our most important discovery, however, is the striking demand for eye care services in this displaced population, despite a host of other competing social and healthcare needs. High proportions of both children (over a third of those aged 5–11 years) and the elderly (nearly two-thirds of those over 60) presented for care at the Orbis-supported eye clinics. It is especially remarkable that the majority of those over 60 years old in the 12 surrounding camps and not only those dwelling in Camps 4 and 11, where the services were delivered and actively advertised, presented for care. This strong demand underscores the important lesson that addressing needs for non-life-threatening conditions, such as vision impairment, may contribute significantly to the improved resiliency necessary for displaced communities to survive and thrive under demanding conditions.

The significantly lower proportion of Rohingya as compared to host child and adult patients requiring glasses for uncorrected refractive error likely reflects the lower burden of myopia generally reported in economically deprived settings [20] and may also indicate a higher prevalence of other, nonrefractive ocular conditions such as infections [21,22], which have been documented in the Rohingya camps.

The existing literature on visual health among displaced populations, including a recent review [11], has documented high prevalence of blindness [12,22–25] in these communities, ranging from 1.3% to 26.2% [11]. However, few published studies [26–29] have focused on the delivery of eye care services rather than simply assessing the existing burden, and none of these have measured the demand for comprehensive eye care service in a defined refugee community, beyond a single disease focus such as refractive error [27–28] or trachoma [29]. The current study also represents the largest to document delivery of and demand for comprehensive eye services in a displaced population.

The high uptake of treatment to ameliorate chronic conditions such as cataract and refractive error is consistent with other recent reports demonstrating significant burden and service demand for conditions such as noncommunicable diseases [30–34] and mental health disorders [5–6] in crisis settings. Unlike these conditions—which require chronic care and whose management may not lead to obvious, subjective improvements that are likely to drive high demand—treatment of eye disease such as cataract leads generally to immediate increase in vision and high patient satisfaction [35]. Good surgical results in a randomly selected sample of consecutive Rohingya patients operated on in our program are consistent with this.

An important part of the service model developed by Orbis and partners in this setting was the delivery of care to the host population as well as the unsettled Rohingya. Consistent with other reports [36], we found a high demand for eye care, particularly eyeglasses, in the host population. Improving vision among children and adults in host communities can help build their capacity to support displaced populations in their midst.

As noted above, strengths of the current report include the large size of the database, careful description of our care model, calculation of age-stratified demand for specific vision care services in this rapidly shifting population, and assessment of service outcome quality, rarely performed [26] for ocular services in crisis settings. Limitations must also be acknowledged. The current report focused on clinic-based assessments, albeit at a variety of community-based settings, and was not designed as a population-based survey. Participants in the current study are very unlikely to be representative of the Rohingya or host populations as a whole, though they are of particular interest from a health services research standpoint. As a result, the actual population burden of cataract and other eye diseases cannot be described here, though we have carried out a population-based Rapid Assessment of Avoidable Blindness in the Rohingya and host communities, which will soon provide such information.

The Rohingya population is shifting between camps for a variety of logistical reasons, and thus the censuses providing the denominators for our demand calculations will inevitably have some accuracy limitations. Our approach of pooling residents across a large number of contiguous camps in most of our tables reduces somewhat the impact of such population shifts. Rohingya patients identified with cataract requiring surgery had the option of being operated on at the Orbis partner clinic CBBSH or another nearby NGO-supported facility (which did not carry out vision screening activities in the areas covered by this report). Thus, we are unable to calculate the acceptance rate for cataract surgery offered in our program. Finally, the data reported here are drawn from several settlement centers and the host community in a single crisis situation involving the Rohingya, and more work in other settings is needed to have confidence in applying these results more broadly to other displaced populations.

### Conclusions

#### Significance for policy makers and public health planning

Significant burdens of vision impairment exist among adults and children in crisis settings, spontaneous demand for services is high, and proven models exist to deliver low-cost, high-quality care to both displaced and host communities in collaboration between NGOs and local partners. These models have the potential for replication elsewhere as the burden of global humanitarian crises continues to grow at an unprecedented rate, due not only to the number of events but also to the prolonged period of displacement, exacerbating the need for management of chronic disease. The burden of treatable eye disease such as cataract, and high demand for sight-restoring service, among adults at the height of the working years is particularly striking, as this cohort contributes very significantly to the resiliency of a displaced community such as the Rohingya.

#### Need for further research

Carefully done studies are needed that demonstrate the potential for vision care to improve the resiliency and quality of life of displaced populations. Existing evidence from settled populations shows that treatment of ocular conditions such as refractive error and cataract can improve children’s educational performance [37], adult workplace productivity [38], and the financial well-being of families [39]. Work is needed to better elucidate the specific implications of these findings for displaced populations, for which enhanced resiliency can be particularly crucial in allowing these challenged communities to thrive.

## Supporting information

S1 STROBE Checklist STROBE, Strengthening the Reporting of Observational Studies in Epidemiology. (DOCX)

## Abbreviations

AIDD: Asian Institute of Disability and Development
BOOST: Better Operative Outcome Software Tool
CBBSH: Cox’s Bazar Baitush Sharaf Hospital
CEITC: Chittagong Eye Infirmary and Training Complex
CiC: Camp in Charge
IOP: intraocular pressure
logMAR: log of the minimum angle of resolution
NGO: nongovernmental organization
PIS: patient information system
STROBE: Strengthening the Reporting of Observational Studies in Epidemiology
UNHCR: United Nations High Commissioner for Refugees
WHO: World Health Organization
